# Translation and Validation of the Dysphagia Handicap Index in Polish-Speaking Patients

**DOI:** 10.1007/s00455-022-10545-y

**Published:** 2022-12-12

**Authors:** Ewelina M. Sielska-Badurek, Maria Sobol, Joanna Chmilewska-Walczak, Barbara Jamróz, Kazimierz Niemczyk

**Affiliations:** 1 Department of Otorhinolaryngology, Head and Neck Surgery, Medical University of Warsaw, Ul. Banacha 1a, 02-097 Warsaw, Poland; 2 The Children’s Memorial Health Institute, Department of Otolaryngology, Audiology and Phoniatrics, Al. Dzieci Polskich 20, 04-736 Warsaw, Poland; 3 Department of Biophysics, Physiology and Pathophysiology, Medical University of Warsaw, Warsaw, Poland

**Keywords:** DHI, Dysphagia Handicap Index, Quality of life, Dysphagia, Oropharyngeal dysphagia, Deglutition, Deglutition disorders

## Abstract

Dysphagia Handicap Index (DHI) is a self-assessment questionnaire which consists of 25 statements to examine three aspects of dysphagia patients’ quality of life (QoL): functional, physical, and emotional. The patient can get a maximum score of 100 points. The study goal was to validate and translate the Polish version of the DHI (PL-DHI). One hundred and seventy-eight (178) individuals with oropharyngeal dysphagia with different etiology and 35 (thirty-five) asymptomatic adults with no history of swallowing disorders filled out the PL-DHI. Internal consistency was determined using Cronbach alpha coefficient, which was high for the total PL-DHI score (0.962). The reproducibility was high (r-Spearman correlation coefficient was 0.97 for total PL-DHI score). The PL-DHI’s total score and its subscales were significantly higher in the dysphagia patients study group (SG) than in the healthy controls group (CG) (SG median: 36; CG median: 4). A strong correlation was observed between the PL-DHI score and the self-reported dysphagia severity measure (Spearman’s correlation coefficient was 0.859, *p* < 0.001). The Polish DHI is a reliable and valid questionnaire for assessing dysphagia patients’ QoL.

## Introduction

Oropharyngeal dysphagia (OD) occurs in 2.3–16% in different populations [1–4]. Moreover, the prevalence of OD increases with age [1]. Actually, OD is presented in 33–40% of the above-60-year-old population and in patients above 75 years in 50% of population; nevertheless in group of elderly community-acquired pneumonia patients, the percentage is higher (91%) [5, 6]. Swallowing disorders are therefore not a marginal problem. We live in an aging society that lives longer and, therefore, is more likely to experience swallowing disorders. The most common causes of swallowing disorders after the age of 60 are neurological diseases (e.g., 8.1–80% of stroke patients, 11–60% of Parkinson’s diseases patients) and head and neck cancers (e.g., 52% patients after radiotherapy, 69% after chemoradiotherapy, 10–72% patients after total laryngectomy, 51% with head and neck cancer patients) [1, 3, 6–8]. The effects of OD range from malnutrition and dehydration, to aspiration pneumonia and asphyxia. These complications can have a significant impact, not only on the health and lives of patients, but also on their quality of life.

Recently, quality of life (QoL) has come to play a key role in treating patients with dysphagia. WHO defines quality of life as “individual perception of their position in life in the context of the culture and value systems in which they live and in relation to their goals, expectations, standards, and concerns” [9]. Furthermore, recently, the self-assessment through patient-centered measures has gained increasing importance, since it may not correlate with clinician-driven tools [10]. Many questionnaires assessing the QoL of patients with dysphagia have been developed. One of these is M.D. Anderson’s Dysphagia Inventory, a questionnaire for Head and Neck Cancer (HNC) patients [11]. However, the Dysphagia Goal Handicap (DGH) was designed for patients with esophageal dysphagia [12]. One of the more general questionnaire is the Swallowing Quality of Life Questionnaire (SWAL-QOL), designed for adult OD patients with etiological heterogeneity [13]. Finally, the Eating Assessment Tool-10 (EAT-10) aims to assess OD-related functional health status [14]. However, for the time being, neither of them has been validated for the Polish language [15]. The Dysphagia Handicap Index (DHI) and the SWAL-QOL are questionnaires for oropharyngeal dysphagia with the strongest psychometric ratings. The SWAL-QOL is considered the gold standard, exhibits good internal consistency reliability and short-term reproducibility, but is longer than the DHI and could be difficult for some groups of patients to fill out [13, 16, 17]. Therefore, the authors chose the DHI Questionnaire to translate and validate because the form is more concise and user-friendly than the SWAL-QOL [18].

The DHI is a self-assessment questionnaire on quality of life as it pertains to and results from the ability to swallow. It consists of 25 statements to which patients match answers according to the three-stage Likert scale, where 0 means "never," 2 means "sometimes," and 4 means "always". These statements examine three aspects of the swallowing disability as it pertains to functional aspects (9 self-assessment questions, numbers: 6, 7, 9, 10, 14, 15, 16, 22, 23), physical condition (9 self-assessment questions, numbers: 1, 2, 3, 4, 5, 11, 20, 24, 25), and emotional state (7 self-assessment questions, numbers: 8, 12, 13, 17, 18, 19, 21). A patient can get a maximum score of 100 points. A score of 0 means the patient is completely satisfied with their ability to swallow. The higher the DHI value, the greater the patient's dissatisfaction with the swallowing quality/ability. Sobol et al. suggest that the normative value of DHI score for a healthy participant is 4, while a score above 4 indicates self-perceived dysphagia symptoms [19]. A visual analogue scale (VAS) is added to the DHI questionnaire. This is a simple scale, commonly used in medicine, to assess the severity of disorders experienced by patients. In the study, the VAS scale was used to help determine the severity of swallowing problems being experienced by the patient. It takes values from 1 to 7. The numbers are graphically represented on a ruler, where 1 is normal swallowing, 4 is a moderate swallowing problem, and 7 is a severe swallowing problem.

DHI is becoming more prevalent, and it has been translated into Hebrew [18], Persian [20], Arabic [21], Japanese [22], and Kannada [23]. It is highly problematic that no questionnaire for examining swallowing-related quality of life has been adapted and validated in Polish language. Since there is no Polish version of the DHI questionnaire (PL-DHI), this study undertakes to translate and adapt it for Polish-speaking population and culture.

## Methods

Approval was obtained from the Polish Local Ethics Committee of the Medical University of Warsaw.

### Translation

**Fig. 1:**
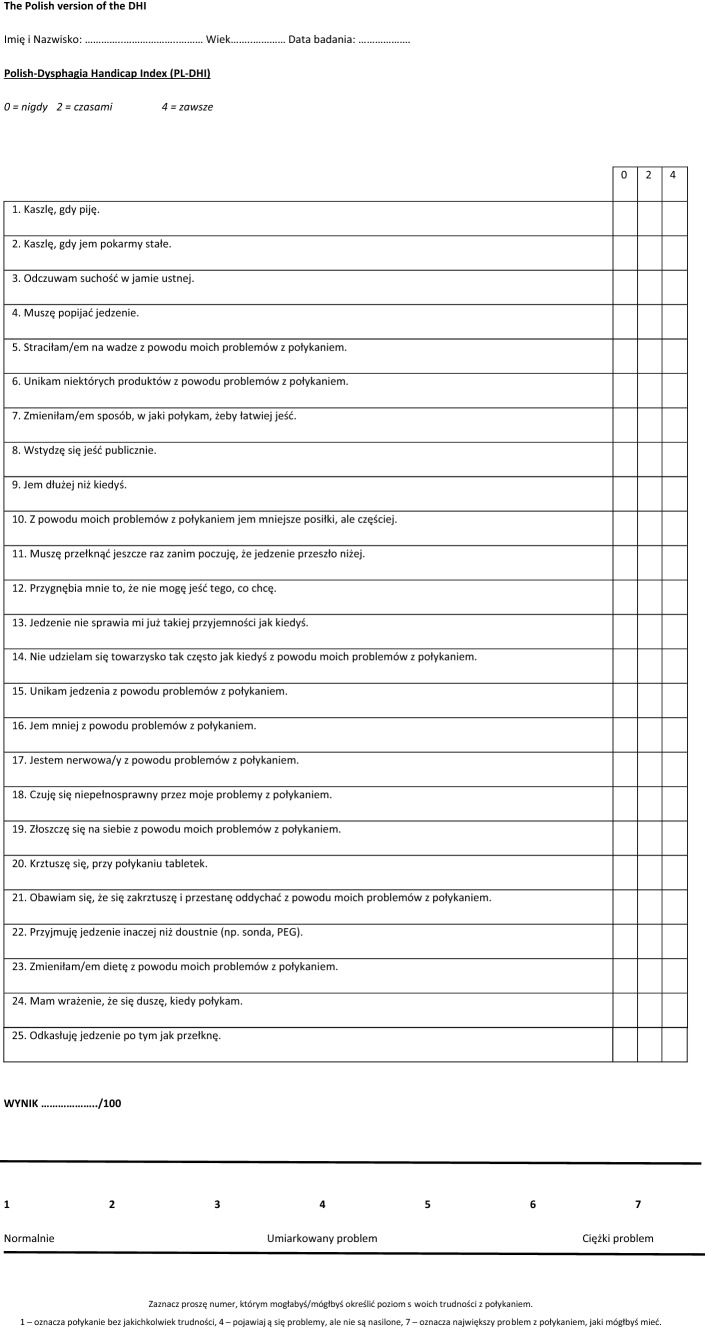
The Polish version of the Dysphagia Handicap Index

In the translation process, the original English version of the DHI was translated along with the principles of good practice carried out for the translation and cultural adaptation process for patient-reported outcome measures as defined by the International Society for Pharmacoeconomics and Outcome Research [24]. The original DHI questionnaire was translated by a speech-language pathologist and Polish philologist who is fluent in English. The translation was discussed with an experienced phoniatrician who is likewise fluent in English and then with a native speaker of English, a qualified professional translator, fluent in Polish. Items of the questionnaire were then back-translated into English and compared against the original DHI. The back-translation (from Polish back into English) was reviewed by the authors. The translation thus reconciled, and which will ultimately be used with Polish patients, was then reviewed and pilot-tested on twenty subjects with oropharyngeal dysphagia with different etiologies from the Speech-Language Pathology and Phoniatrics Clinic at the Medical University of Warsaw. The next step was to assess internal consistency using the Cronbach's alpha coefficient (Cronbach's *α*). The correctness of the test was determined using the Spearman rank correlation coefficient. No major corrections were made, and the results were the PL-DHI (Fig. 1.).

### Procedure

Inclusion criteria in the study group were (1) presence of OD deriving from any etiology, (2) age over 18, and (3) ability to independently read a written text. Dysphagia was defined as eating disturbance of the intake or transport of food from the month to the stomach. Oropharyngeal dysphagia was defined as disturbance during oral preparatory, oral transport, or pharyngeal phase of swallowing. Moreover, if there were residuals (including those in the oral cavity) that increased the risk of aspirations, we defined these subjects as OD patients [25].

Exclusion criteria were (1) inability to understand written Polish, (2) cognitive dysfunction (we excluded subjects with poor logical and verbal contact or any with cognitive dysfunction mentioned in neurological medical documentation), or (3) evidence of purely esophageal dysphagia.

After a short oral instruction (given each time by the first person—SLP), participants filled in independently questionnaire using paper and pencil, on the same day without later help. The oral instruction included a request to fill in a questionnaire by assigning three responses for each question (never, sometimes, and always), adding a value to each response (0, 2, and 4, respectively). Moreover, each participant was asked to self-rate the severity of their dysphagia on a 7-point equal appearing interval scale (visual analogue scale—VAS) anchored by the number 1 and the word ‘‘normal’’ on one end, the number 7 and the word ‘‘severe problem’’ at the other end, and the number 4 in the middle indicating a moderate swallowing problem. In the study group, patients filled in the DHI—Questionnaire after FEES examination. All participants included in the study were able to read and complete the DHI Questionnaire independently.

The patients were qualified into the study after clinical swallowing examination (carried out by SLP) and FEES examination (carried out by phoniatrician and SLP, trained during FEES Accreditation Courses) [26]. Both examinations were performed within the same week. FEES was performed using a XION flexible endoscope with a chip on tip camera and a 4 mm diameter. Swallowing was evaluated directly with nine bolus challenges, five of each consistency (liquid, puree, and solid) of approximately 2 cc volume, followed by 5 cc volume and a series of three times 5 cc volume each. The consistencies were presented as follows: five boluses of puree consistency (blue-dyed apple puree) followed by a solid consistency challenge of whole wheat bread (five pieces) and concluded with five thin liquid boluses (blue-dyed water). Patients were encouraged to feed themselves, with assistance as needed, i.e., liquid with a straw or cup and puree with a spoon. If either consistency or volume was considered unsafe to be administered or if severe swallowing efficacy impairment was observed, the FEES protocol was not completed with unsafe volumes or consistencies. Owing to safety reasons, the FEES protocol was interrupted if at least one of the following conditions occurred: (1) severe impairment of the oral control of the bolus with pureed food which led to chocking (2) and severe impairment of the oral preparatory swallowing stage with solids which prevented the processing of the solid into a bolus.

The presence and degree of airway invasion were measured using the penetration–aspiration scale (PAS) [27, 28]. Penetration was scored as present with PAS ≥ 3 ≤ 5 [29], while aspiration with PAS ≥ 6 [29] and all these patients were included in the study group. The worst bolus (i.e., the bolus with the highest PAS score) for each consistency tested and the worst PAS score among all consistencies were considered for the analysis.

### Statistical Analysis

A statistical analysis was performed using the program Statistical13. For the quantitative variables, the scores were summarized using descriptive statistics (mean, standard deviation, median, and range). The reliability of the DHI was determined, examining the internal consistency and test–retest reliability. The internal consistency of the total DHI and physical, functional, and emotional subscales was evaluated using the Cronbach alpha coefficient. The distribution of each quantitative variable was checked for consistency against the normal distribution (Shapiro–Wilk test). The number of samples available for the test–retest reproducibility analysis determined the application of the *r*-Spearman coefficient. The nonparametric Kruskal–Wallis test was used to compare the DHI scores in the four subgroups with swallowing disorders and CG. To assess the differences between them, the nonparametric Mann–Whitney test was performed. Because of multiple comparisons, Bonferroni correction was included. The results were considered statistically significant if the *p* value was less than 0.05 (*p* < 0.05) or 0.02 (*p* < 0.017) in the case of multiple comparisons.

## Results

### Participants

The study group was recruited from the Department of Otolaryngology at the Medical University of Warsaw from March 2016 to June 2018. Initially, 191 patients were considered for inclusion in the study group. Thirteen patients were excluded from the study (OD was excluded in 6 patients, 3 patients with the cerebrovascular bleeding presented with poor logical and verbal contact, 2 patients presented with neurofibromatosis type 2 due to amblyopia and hearing loss were unable to complete the questionnaire, and 2 patients refused to participate in the study due to malaise after the neurosurgical intervention). Finally, 178 subjects with oropharyngeal dysphagia were included in the study. The patients presented with a wide range of diagnoses, and different etiologies of swallowing disorders, including neurological disorders—study group I (stroke, Alzheimer’s, myasthenia gravis, mitochondrial myopathy), head and neck cancer—study group II (paragangliomas, free flap reconstruction, strumectomy, vocal fold paresis, partial laryngectomy), neurosurgical operations—study group III (cerebellopontin angle tumor, brain tumors), and other disorders including LPR (laryngopharyngeal reflux disorder), gastrointestinal tract disorder, chronic cough, Zencer’s diverticulum and post-cardiothoracic surgeries—study group IV (Table 1).

**Table 1:** Demographic characteristic

	Gender *N* (%)	Diagnosis *N* (%)	Age		
			Mean ± SD	Median	Range
SG *n* = 178	F 103 (57.9%) M 75 (42.1%)	Paraganglioma 37 (20.8%) Larynx carcinoma 19 (10.7%) Parotidectomy 18 (10.1%) Gastroesphagel Reflux Disease 14 (7.9%) Skull base tumor 9 (5.1%) Tongue and floor of the mouth carcinoma 9 (5.1%) Thyroidectomy 9 (5.1%) Stroke 9 (5.1%) Parapharyngeal space tumor 7 (3.9%) Degenerative changes of the cervical spine 5 (2.8%) Myopathy 5 (2.8%) Parkinson's disease 4 (2.2%) Benign lesion of the larynx 4 (2.2%) Obstructive sleep apnea 3 (1.7%) Tongue carcinoma 3 (1.7%) Amyotrophic lateral sclerosis 2 (1.1%) Myasthenia gravis 2 (1.1%) embolization of brain stem hemangiomas 1(0.6%) Others ^abc^—18	56.8 ± 15.0	59	21–91
CG *n* = 35	F 21 (60%) M 14 (40%)		52.6 ± 12.3	51	23–88
SG I *n* = 28	F 15 (53.6%) M 13 ( 46,4%)	Stroke 9 (32%) Myopathy 5 (17.9%) Parkinson's disease 4 (14.3%) Myasthenia gravis 2 (7.1%) Amyotrophic lateral sclerosis 2 (7.1%) Others^a^ 6 (17.9%)	55.7 ± 15.1	60	26–87
SG II *n* = 106	F 59 (55.7%) M 47 (44.3%)	Paraganglioma 37 (35%) Larynx carcinoma 19 (17.9%) Parotidectomy 18 (17%) Thyroidectomy 9 ( 8.5%) Tongue and floor of the mouth carcinoma 9 (8.5%) Parapharyngeal space tumor 7 (6.6%) Tongue carcinoma 3 (2.8%) Others^b^ 4 ( 3.6%)	56,9 ± 13.7	59	21–83
SG3- III *n* = 10	F 7 (70%) M 3 (30%)	Skull base tumor 9 (90%) Embolization of brain stem hemangiomas 1 (10%)	47.5 ± 18.0	55.5	25–68
SG IV *n* = 34	F 22 (64.7%) M 12 (35.3%)	Gastroesphagel Reflux Disease 14 (41%) Degenerative changes of the cervical spine 5 (14.9%) Benign lesion of the larynx 4 (11.8%) Obstructive sleep apnea 3 (8.8%) Others^c^ 8 ( 23.3%)	59.6 ± 17.3	61.5	21–91
SG Test–retest reliability *n* = 24	F 13 (45.8%) M 11 (54.2%)	Paraganglioma 6 (25%) Skull base tumor 6 (25%) Stroke 3 (12.5%) Tongue and floor of the mouth carcinoma 2 ( 8.3%) Others^d^ (29.2%)	53.9 ± 16.2	55	25–83

SG—study group; CG—control group; SG—I—study group I—neurological disorders; SG—II—study group II- head and neck cancer; SG—III—study group III—neurosurgical operations; SG IV- study group IV- other disorders, F—female, M- male

^a^Mitochondrial encephalopathy, Dystonia, polyneuritis, supra-nuclear paralysis, dystrophy

^b^Larynx and hypopharynx carcinoma, pharynx carcinoma, floor of the mouth carcinoma

^c^Thyroidectomy, esophageal resection with gastro-esophageal anastomosis, Lip/cleft palate, Zencker diverticulum, lymphangioma, schatzki ring

^d^Amyotrophic lateral sclerosis, obstructive sleep apnea, parapharyngeal space tumor, Parkinson's disease, parotidectomy, multi-muscle lesion, embolization of brain stem hemangiomas"

The control group consisted of 35 asymptomatic adults who had no history of any swallowing disorders or LPR, no history of head and neck surgeries, no other risk factors for oropharyngeal dysphagia, and no other chronic diseases.

The control group was recruited from individuals accompanying patients, as well as hospital staff and their family members. PL-DHI was given to everyone from the recruited control group over a period of two weeks (between 13 and 14 days). During this period, the 35 people comprising the control group did not have any swallowing intervention or medical or surgical intervention. All participants were Caucasian. Table 1 shows the demographic characteristic of SG and CG.

In analysis, we considered only patients without missing data. Table 2 shows the mean value, standard deviation (SD), median, and the range of the PL-DHI scores of the SG and subgroups and the CG. Table 3 presents the distribution of the DHI subscales and DHI total scores for SG. It is important to notice that in the control group, one patient was scored an 88 on the DHI. In this patient, VFSS examination was performed to exclude the swallowing disorders, objectively.

**Table 2:** Value of PL -DHI for patients and control group

	PL-DHI		
	Mean ± SD	Median	Range
SG	40.1 ± 27.2	36	0–100
CG	14.2 ± 23.7	4	0–96
SG I	53.1 ± 24.0	48	22–100
SG II	36,5 ± 27.8	28	0–98
SG III	45.6 ± 21.6	34	26–94
SG IV	37.7 ± 26.8	39	4–90

**Table 3:** Distribution of the PL-DHI subscales and PL-DHI total scores for SG

DHI scale *n* = 178	No. items	Mean ± SD	Median (Min–max)
Total	25	40.1 ± 27.2	36 (0–100)
Physical	9	15.5 ± 9.2	16 (0–36)
Functional	9	14.3 ± 10.6	14 (0–36)
Emotional	7	10.8 ± 9.3	8 (0–28)

In the study group, swallowing safety was scored as a PAS of 3 (range 1–8). In SG I, patients scored a PAS of 2.5 (range 1–8), in SG II—3 (range 1–7), in SG III—4 (range 1–8), and in SG IV—1 (range 1–7).

### Internal Consistency

The internal consistency of the DHI was determined using the Cronbach alpha coefficient, with values between 0.7 and 0.8 considered as acceptable, 0.8–0.9 considered as good and values higher than 0.9 were considered to be excellent (strong). The internal consistency for the total DHI score was 0.962. A more detailed analysis of each question indicates that all of them have a similar influence on the reliability of the overall scale. The Alpha coefficient for items 1–25 ranged from 0.959 (for the item no. 14) to 0.963 (for item no. 3). The Cronbach’s alpha coefficients were also high for the DHI subscales: physical, functional, and emotional, which came in, respectively, at 0.878, 0.896, and 0.0,898. A strong Cronbach alpha coefficient, as in the study, indicates that the items are measuring the same construct (Table 4).

**Table 4:** Internal consistency—Cronbach's alpha results reported for each item of the DHI

Item (number)	Standardized α-Cronbach a, 0.955				
	Scale mean if item delated	Scale variance if item delated	Scale SD if item delated	Item—total correlation	Cronbach’s alpha if item delated
1	37.6	721.1	26.853	0.629	0.956
2	37.6	717.0	26.777	0.680	0.956
3	37.0	740.9	27.219	0.302	0.963
4	36.8	721.3	26.857	0.467	0.958
5	38.2	714.9	26.738	0.590	0.957
6	37.3	697.3	26.407	0.775	0.955
7	37.3	706.0	26.571	0.653	0.956
8	37.7	697.7	26.414	0.725	0.955
9	36.7	708.3	26.614	0.634	0.956
10	37.4	701.4	26.483	0.712	0.955
11	37.2	698.6	26.432	0.782	0.955
12	37.7	698.9	26.436	0.751	0.955
13	37.6	697.6	26.412	0.753	0.955
14	37.7	690.1	26.269	0.817	0.954
15	38.1	704.8	26.549	0.781	0.955
16	37.7	702.5	26.505	0.769	0.955
17	37.8	701.4	26.484	0.744	0.955
18	38.0	703.7	26.528	0.727	0.955
19	37.9	705.9	26.568	0.715	0.955
20	37.5	699.0	26.439	0.750	0.955
21	37.6	708.3	26.614	0.661	0.956
22	38.7	741.1	27.223	0.370	0.958
23	37.8	707.7	26.603	0.670	0.956
24	38.2	715.5	26.748	0.675	0.956
25	37.7	712.3	26.689	0.684	0.956

### Test–Retest Reliability

In order to assess DHI test–retest reliability, a randomly selected subsample of 24 subjects completed the DHI a second time, 13–14 days after the initial assessment (Table 1 presents the demographic of the study group selected for the test–retest reliability).

Test–retest reliability scores are satisfactory for total score and all DHI-PL subscales. ICC values less than 0.5 indicate poor reliability, moderate from 0.5 to 0.75, good from 0.75 to 0.9, and excellent reliability if values are greater than 0.9. The ICCagreement values for the control group were 0.974 with confidence interval CI (0.948–0.987) for the physical subscale, 0.992 for functional subscale with 0.983–0.996 CI, for emotional subscale 0.988 with 0.976–0.994 CI and 0.993 with 0.986–0.996 for the Total score. Moreover, for 24 subjects of the study group, the ICCagreement values were 0.955 with confidence interval CI (0.897–0.981) for the physical subscale, 0.857 for functional subscale with 0.670–0.938 CI, for emotional subscale 0.944 with 0.871–0.976 CI, and 0.968 with 0.927–0.986 for the total score, which indicates excellent reliability for all the DHI subscales except good reliability for functional subscale in study group. In addition, we received excellent reliability of total score in both groups.

**Fig. 2:**
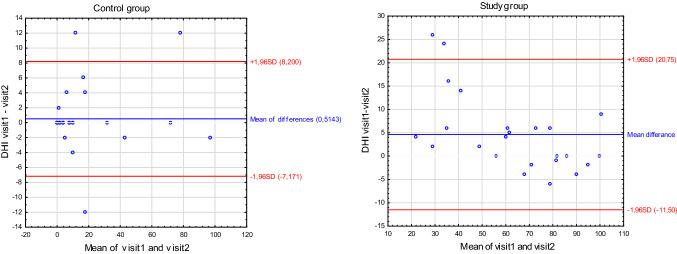
Bland–Altman plot–control and study groups

To measure test–retest reliability in the control group, the DHI was completed and sent or brought back by the 35 participants twice within a two-week period. The median and the range of the total score of DHI were 4 (0–96) and 4 (0–98), respectively, for the DHI first and second assessments. To measure the test–retest reproducibility, the Spearman range test was used. The *r*-Spearman correlation coefficient for control group was *r* = 0.97 for the total score of DHI. For the DHI physical, functional, and emotional subscales, the *r*-Spearman coefficients were, respectively, 0.91, 0.86, and 0.83. This indicates a very good level of reproducibility. In case of study group, *r* = 0.97 for the total score of DHI. For the DHI physical, functional, and emotional subscales, the *r*-Spearman coefficients were, respectively, 0.90, 0.77, and 0.88. Figure 2 presents the correlations between the first and second assessment in control and study groups.

### DHI-SubScales correlation

The DHI subscale scores were not normally distributed (Shapiro–Wilk test), so the correlations between the subscales were assessed using the *r*-Spearman correlation test (very strong correlation 0.9–1, strong 0.7–0.89, moderate 0.4–0.69, weak 0.1–0.39, and no correlation 0–0.09). The correlation was highest between the emotional and functional subscales (*r*-Spearman coefficient 0.835) and lowest between the physical and emotional subscales (*r*-Spearman coefficient 0.792). The correlation coefficient between the functional and physical was 0.834.

### Construct Validity Analysis (Discriminant Validity)

**Fig. 3:**
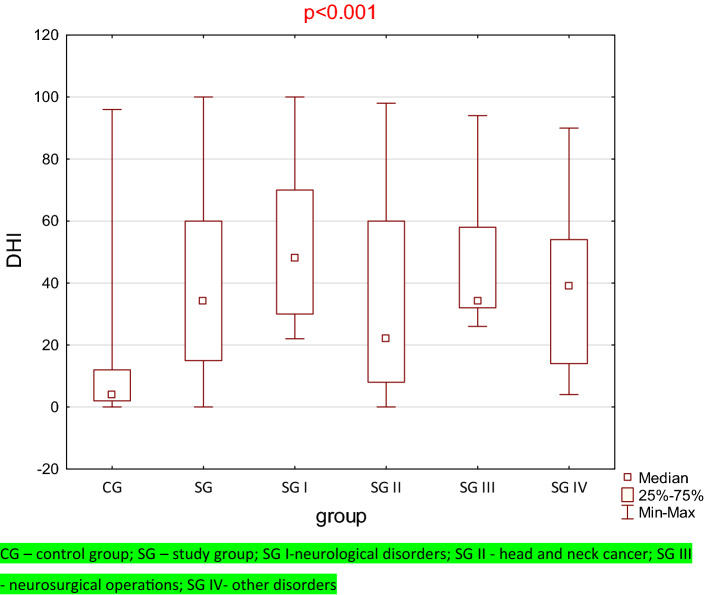
Box plot for the control group, entire study group and for each subgroup with DHI results

**Fig. 4:**
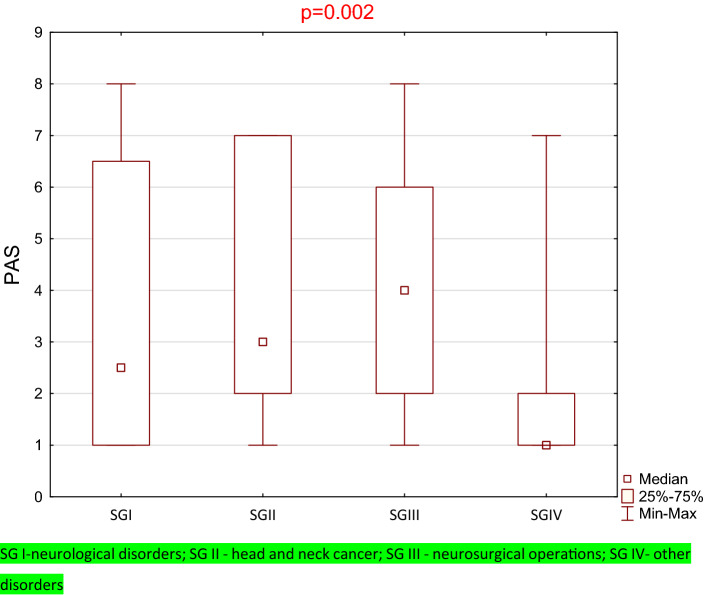
Box plot for each subgroup with PAS results

The results of the Kruskal–Wallis analysis of variance for DHI showed a statistically significant difference for the considered subgroups (*p* < 0.001) (Fig. 3). The differences in multiple comparisons (between the CG and patients from groups I, II, III, and IV) for DHI were analyzed using a nonparametric Mann–Whitney test followed by Bonferroni correction. The results were considered statistically significant when the *p* value was less than 0.017. Significant differences were found between the CG and SG I to SG IV (*p* < 0.001). Moreover, the Mann–Whitney test was used to check whether there were statistically significant differences between the subgroups of the study group (SG). Due to multiple comparisons, Bonferroni corrections were applied, and the level significance was assumed as *α* = 0.008. Statistically significant differences were only found between SG I and SG II (*p* < 0.001) (Fig. 4).

For swallowing safety score, we received statistically significant differences between subgroups (Kruskal–Wallis test). Moreover, the Mann–Whitney test was used to check whether there were statistically significant differences between the subgroups of the study group (SG). Due to multiple comparisons, Bonferroni corrections were applied, and the level of significance was assumed as *α* = 0.003. Statistically significant differences were found only between SG II and SG IV (*p* < 0.001) (Table 5).

**Table 5:** PAS scores in subgroups of study group and comprehension of PAS scores between subgroups of the SG

	PAS			
	SG I	SG II	SG III	SG IV
	*n* = 28	*n* = 80	*n* = 9	*n* = 28
Median	2.5	3	4	1
(Min–Max) IQR	(1–8) 5.5	(1–7) 5	(1–8) 4	(1–7) 1

	*p*- value *U*-Mann–Whitney test			
SG I		0.231	0.453	0.453
SG II	0.231		0.909	< 0.001
SG III	0.453	0.909		0.053
SG IV	0.453	< 0.001	0.053	

*IQR* the interquartile range

*SG I* neurological disorders; *SG II* head and neck cancer; *SG III* neurosurgical operations; *SG IV* other disorders

**Fig. 5:**
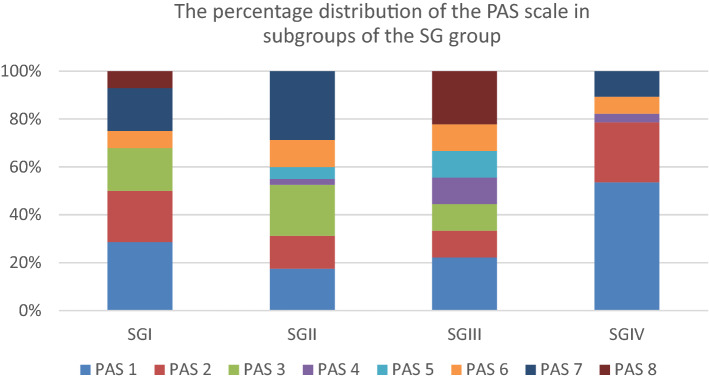
The percentage distribution of PAS scores for each subgroup of the SG group

Regardless of the consistency tested, 39 (26.9%) patients presented no sign of aspiration or penetration, 57 (39.3%) showed penetration (PAS 2–5), and 49 (33.8%) showed aspiration (PAS 6–8). The percentage distribution of PAS scores for each subgroup of the SG group is given in Fig. 5.

### Criterion Validity Analysis

We received statistically significant weak positive correlation between swallowing safety rated through the worst PAS score and physical (*r*_*S*_ = 0.205 *p* = 0.013) functional (*r*_*S*_ = 0.266 *p* = 0.001), and emotional (*r*_*S*_ = 0.182 *p* = 0.029) subscales and total score of DH I(*r*_*S*_ = 0.243 *p* = 0.003).

### Correlations

The results’ correlation of the DHI total score and VAS value is presented in Table 6. There was noticed high positive correlation between the DHI total score and VAS.

**Table 6:** R-Spearman coefficient between total PL-DHI score and VAS

Group	Number of patients	R-Spearman coefficient	*p* value
CG + SG	213	0.859	< 0.001
SG	178	0.812	< 0.001
CG	35	0.720	< 0.001

*CG* control group, *SG* study group, *PL-DHI* Polish Version of the Dysphagia Handicap Index, *VAS* visual analogue scale

## Discussion

In the last few years, the quality of life related to swallowing and voice has received a great deal of attention. But we still do not have a validated questionnaire in the Polish language to evaluate QoL related to swallowing. Focusing on that, the purpose of this study was to evaluate the validity and reliability of the PL-DHI. The number of subjects in the SG (*n* = 187) may be seen as a strength of the present study.

The results revealed that the PL-DHI is a reliable tool with good internal consistency and test–retest reliability. These results are similar to those for the original DHI and to the translation of DHI into other languages [16, 18, 20–22].

The Cronbach’s alpha coefficient for the total PL-DHI and physical, emotional and functional subscales was between 0.864 and 0.955, indicating that the PL-DHI had good internal consistency. Alpha values lower than 0.70 reflect a low correlation among the instrument items and may suggest an insufficient or poorly chosen set of items. The obtained α values could be interpreted as satisfactory.

PL-DHI internal consistency, and thus no DHI items were inserted nor deleted.

The median score for the SG was 36, with the highest SG I of 48 points in neurological patients. The SG median is significantly higher than the control group median (CG—4 points). Based on the PL-DHI score, it is possible to discriminate between healthy controls and OD patients. However, one patient in study group was scored with 0, although the presence of OD features in FEES examination. It proves that the questionnaire is still a subjective diagnostic tool. The normative data generated in the present study align with the DHI normative values through a systemic review and meta-analysis developed by Sobol et al., who reported 2.49 (0.51–4.48) as DHI mean [19].

The mean value score in PL-DHI was significantly higher (40.1 ± 27.2) than in original DHI (27.33 ± 21.18) but very similar to the Hebrew DHI (38.44 ± 24.39). Comparison of the different translations reveals a wide range of score. Table 7 presents a comparison among data of different DHI translation studies. The Japanese DHI [22] is markedly lower than all others. As Shapira [18] observed, this may be attributable to the cultural differences in the self-appreciation of dysphagia severity or to differences between the populations tested. Additionally, Ginocchio underlines that the significantly higher DHI scores reported by OD patients compared to healthy participants adequately reflect the impact of OD on patients’ health-related quality of life [30].

**Table 7:** Comparison among data of different DHI translation studies

	Polish DHI	Hebrew DHI [6]	DHI [6]	Persian DHI [9]	Arabic DHI [10]	Japanese DHI [11]	Kannada [12]
Total DHI	36 (0–100) 40.1 ± 27.2	39 (18–56) 38.44 ± 24.39	– 27.33 ± 21.18	28 (0–92) 32.14 ± 25.32	– 32.489 ± 24.737	10 (2–24) –	– 77.72 ± 21.25
Physical	16 (0–36) 15.5 ± 9.2	14 (10–20) 14.39 ± 8.16	– 11.52 ± 6.86	11 (0–33) 15.23 ± 7.97	– 13.3 ± 9.6	4 (0–10) –	– 27.13 ± 7.99
Functional	14 (0–36) 14.4 ± 10.6	12 (4–20) 13.38 ± 9.94	– 10.04 ± 9.79	12 (0–34) 10.19 ± 10.86	– 12.3 ± 10.1	2 (0–8) –	– 28.04 ± 8.02
Emotional	8 (0–28) 10.8 ± 9.3	8 (2–16) 10.09 ± 8.57	– 5.76 ± 6.78	4 (0–28) 6.53 ± 5.76	– 6.9 ± 7.4	2 (0–8) –	– 22.59 ± 5.81

The values presented are either mean ± standard deviation or median and interquartile range, or both when available

For the SG in subscales: physical, functional, and emotional, the physical domain score was higher than the functional and emotional ones. Similar results were observed in the translation of DHI to other languages [16, 18, 20–22]. This tendency may result from the fact that symptoms from the physical subscale, including coughing, choking, and unintentional weight loss, may have the greatest impact on the quality of life.

Consistently with the Italian validation study [30], PL-DHI was weakly associated with swallowing safety.

### Limitations and Future Directions

It is important to acknowledge the limitation of the assessment process. Some studies have correlated this questionnaire with other quality of life questionnaires. This study has not done that, which can be considered as limitation.

Moreover, the self-assessment questionnaire performed in this study could be correlated with FEES or VFSS which are the gold standard for diagnosis of oropharyngeal dysphagia. Such comparative studies should be undertaken in future.

Due to the diverse etiology of swallowing disorders, the division of subjects into subgroups for the purposes of the study was difficult. We considered other divisions, but none were homogeneous. Particularly, the study group 4 should be divided into smaller subgroups in future studies. There is a need to conduct further research concerning differentiation between head and neck cancer patients who received chemoradiation and those who did not, as well as those who were diagnosed with vocal fold paresis after head and neck surgery or underwent partial laryngectomy. Those patients were included in one subgroup on the basis of FEES evaluation.

Also, the study was designed at the end of 2015, so we used the Classical Test Theory to validate the PL- DHI, although, nowadays, the Item Response Theory is superior to it in validating patient-reported outcome measures [31]. Consequently, responsiveness and floor ceiling effects were not evaluated. In addition, we examined only construct validity, content, and criterion validity that were not evaluated too.

Future studies are required to validate the PL-DHI using the Item Response Theory and to examine the responsiveness of the DHI after implementation of behavioral strategies, and medical or surgical interventions.

Lastly, the cognitive decline was not measured with any scale, i.e., Mini Mental State Examination. Using this scale would objectify the exclusion criteria.

## Conclusion

Our study demonstrates that the PL-DHI maintained its validity and reliability as a self-assessment tool for oropharyngeal dysphagia patients’ QoL for a Polish-speaking population.

This means that PL-DHI, as an easy-to-complete tool for assessing the consequences of dysphagia on the QoL, is useful not only for researchers, but also for above all patients in a clinical setting.

## References

[1] Bramanti E, Arcuri C, Cecchetti F, Cervino G, Nucera R, Cicciu M (2012). Dental managment in dysphagia syndrome patients with previously acquired brain damages. Dental Res J.

[2] Warnecke T, Dziwas R, Langmore S (2021). Neurogenic dysphagia.

[3] Kertscher B, Speyer R, Fong E, Georgiou AM, Smith M (2015). Prevalence of oropharyngeal dysphagia in the Netherlands: a telephone survey. Dysphagia.

[4] Wolf U, Eckert S, Walte G, Wienke A, Barte S (2021). Prevalence of oropharyngeal dysphagia in geriatric patients and real-life associations. Sci Rep.

[5] Garcia-Peris P, Parón L, Velasco C, de la Cuerda C, Camblo M, Breton I, Herencia H, Verdaguer J, Navarro C, Clave P (2007). Long-term prevalence of oropharyngeal dysphagia in head and neck cancer patients: Impact on quality of life. Clin Nutr.

[6] Takizawa C, Gemmell E, Kenworthy J, Speyer R (2016). A Systematic review of prevalence of oropharyngeal dysphagia in stroke, Parkinson's disease, Alzheimer's disease, head injury, and pneumonia. Dysphagia.

[7] Czernuszeno A (2016). Postępowanie w dysfagii neurogennej. Otolaryngologia.

[8] Baijens LW, Walshe M, Aaltonen LM (2020). European white paper: oropharyngeal dysphagia in head and neck cancer. Eur Arch Oto-Rhino-Laryngol.

[9] World Health Organization (1997). The World Health Organization Quality of Life instruments. Measuring the Quality of Life.

[10] Speyer R (2013). Oropharyngeal dysphagia screening and assessment. Otolaryngol Clin N Am.

[11] Chen AY, Frankowski R, Bishop-Leone J, Hebert T, Leyk S, Lewin J, Goepfert H (2001). The development and validation of a dysphagia-specific quality-of-life questionnaire for patients with head and neck cancer anderson dysphagia inventory. Arch Otolaryngol Head Neck Surg.

[12] Gustafsson B, Tibbling L (1991). Dysphagia, an unrecognized handicap. Dysphagia.

[13] McHorney C, Robbina J, Lomax K, Rosenbek JC, Chingnell K, Kramer AE, Bricker E (2002). The SWAL_QOL and SWAL_CARE Outcomes Tool for Oropharyngeal Dysphagia in Adults: III. Documentation of Reliability and Validity. Dysphagia.

[14] Belafsky PC, Mouadeb DA, Rees CJ, Pryor JC, Postma GN, Allen J, Leonard RJ (2008). Validity and Reliability of the Eating Assessment Tool (EAT-10). Ann Otol Rhinol Laryngol.

[15] Chen AY, Frankowski R, Bishop-Leone J, Hebert T, Leyk S, Lewin J, Goepfert H (2001). The development and validation of a dysphagia-specific quality-of-life questionnaire for patients with head and neck cancer: the M. D. Anderson dysphagia inventory. Arch Otolaryngol Head Neck Surg.

[16] Silbergleit AK, Schultz L, Jacobson BH, Beardsley T, Johnson AF (2012). The dysphagia handicap index: development and validation. Dysphagia.

[17] Speyer R (2013). Oropharyngeal dysphagia. Otolaryngol Clin N Am.

[18] Shapira-Galitz Y, Drendel M, Yousovich-Ulriech R, Shtreiffler-Moskovich L, Wolf M, Lahav Y (2018). Translation and validation of the dysphagia handicap index in hebrew-speaking patients. Dysphagia.

[19] Sobol M, Kober AM, Sielska-Badurek EM (2021). The Dysphagia Handicap Index (DHI)—normative values. Systematic review and meta-analysis. Dysphagia.

[20] Asadollahpour F, Baghban K, Asadi M (2015). Validity and reliability of the persian version of the Dysphagia Handicap Index (DHI). Iran J Otorhinolaryngol.

[21] Malki KM, Mesallam TA, Bukhari M, Alharethy S, Farahat M (2014). Development of the Arabic version of Dysphagia Handicap Index (DHI). Dysphagia.

[22] Oda C, Yamamoto T, Fukomoto Y, Nakayama K, Sato M, Murata M, Kobayashi Y (2017). Validation of the Japanese translation of the Dysphagia Handicap Index. Patient Prefer Adherence.

[23] Krishnamurthy R, Balasubramanium RK (2020). Translation and Validation of Kannada Version of the Dysphagia Handicap Index. Am J Speech Lang Pathol.

[24] Wild D, Grove A, Martin M, Eremenco S, McElroy S, Verjee-Lorenz A, Ericson P (2005). Principles of good practice for the translation and cultural adaptation process for patient-reported outcome (PRO) measures: report of the ISPOR task force for translation and cultural adaptation. Value Health.

[25] Denk-Linnert DM, Ekberg O (2019). Evaluation of symptoms. Dypshagia diagnosis and treatment.

[26] Dziewas R, Baijens L, Schindler A, Verin E, Michou E, Clave P (2017). European Society for Swallowing Disorders FEES Accreditation Programm for Neurogenic and geriatric oropharyngeal dysphagia. Dysphagia.

[27] Rosenbek JC, Robbins JA, Roecker EB, Coyle JL, Wood JL (1996). A penetration-aspiration scale. Dysphagia.

[28] Colodny N (2002). Interjudge and intrajudge reliabilities in fiberoptic endoscopic evaluation of swallowing (fees) using the Penetration-Aspiration Scale: a replication study. Dysphagia.

[29] Rosenbek JC, Robbins JA, Roecker EB (1996). A penetration-aspiration scale. Dysphagia.

[30] Ginocchio D, Ninfa A, Pizzorni N, Lunetta C, Sansone VA, Schindler A (2021). Cross-Cultural Adaptation and Validation of the Italian Version of the Dysphagia Handicap Index (I-DHI). Dysphagia.

[31] Speyer R, Cordier R, Bouix C (2022). Using classical test theory to determine the psychometric properties of the Deglutition Handicap Index. Dysphagia.

